# GW501516-activated PPARβ/δ promotes liver fibrosis via p38-JNK MAPK-induced hepatic stellate cell proliferation

**DOI:** 10.1186/2045-3701-2-34

**Published:** 2012-10-10

**Authors:** Radina Kostadinova, Alexandra Montagner, Erwan Gouranton, Sébastien Fleury, Hervé Guillou, David Dombrowicz, Pierre Desreumaux, Walter Wahli

**Affiliations:** 1 Center for Integrative Genomics, National Research Center Frontiers in Genetics, University of Lausanne, Genopode Building, 1015, Lausanne, Switzerland; 2 Present address: Hoffmann-La Roche AG, Grenzacherstrasse 124, 4070, Basel, Switzerland; 3 INRA ToxAlim, Integrative Toxicology and Metabolism, UMR1331, 180, Chemin de Tournefeuille, BP 93173, 31027, Toulouse Cedex 3, France; 4 Inserm U1011, Institut Pasteur de Lille, 1, University of Lille Nord, rue Prof. Calmette, BP245, 59019, Lille Cedex, France; 5 Inserm U 995, Department of Gastroenterology CHU Lille, University of Lille Nord, 59019, Lille Cedex, France

**Keywords:** Peroxisome proliferator-activated receptor β/δ, Inflammation, Fibrosis, Signaling pathways, Proliferation

## Abstract

**Background:**

After liver injury, the repair process comprises activation and proliferation of hepatic stellate cells (HSCs), which produce extracellular matrix (ECM) proteins. Peroxisome proliferator-activated receptor beta/delta **(**PPARβ/δ) is highly expressed in these cells, but its function in liver repair remains incompletely understood. This study investigated whether activation of PPARβ/δ with the ligand GW501516 influenced the fibrotic response to injury from chronic carbon tetrachloride (CCl_4_) treatment in mice. Wild type and PPARβ/δ-null mice were treated with CCl_4_ alone or CCl_4_ co-administered with GW501516. To unveil mechanisms underlying the PPARβ/δ-dependent effects, we analyzed the proliferative response of human LX-2 HSCs to GW501516 in the presence or absence of PPARβ/δ.

**Results:**

We found that GW501516 treatment enhanced the fibrotic response. Compared to the other experimental groups, CCl_4_/GW501516-treated wild type mice exhibited increased expression of various profibrotic and pro-inflammatory genes, such as those involved in extracellular matrix deposition and macrophage recruitment. Importantly, compared to healthy liver, hepatic fibrotic tissues from alcoholic patients showed increased expression of several PPAR target genes, including phosphoinositide-dependent kinase-1, transforming growth factor beta-1, and monocyte chemoattractant protein-1. GW501516 stimulated HSC proliferation that caused enhanced fibrotic and inflammatory responses, by increasing the phosphorylation of p38 and c-Jun N-terminal kinases through the phosphoinositide-3 kinase/protein kinase-C alpha/beta mixed lineage kinase-3 pathway.

**Conclusions:**

This study clarified the mechanism underlying GW501516-dependent promotion of hepatic repair by stimulating proliferation of HSCs *via* the p38 and JNK MAPK pathways.

## Background

Chronic liver disease represents an important cause of mortality and morbidity. Repeated and/or chronic injury exacerbates wound healing and tissue remodeling processes, leading to progressive fibrosis and, ultimately, end-stage cirrhosis. Currently, the only effective treatment for end-stage cirrhosis is liver transplantation [1]. Therefore, therapeutic interventions that block early stage progression of hepatic fibrosis are important for the prevention of liver cirrhosis. In wounded areas, HSCs are stimulated by factors that promote proliferation and transition from a quiescent, lipid/vitamin A-storing phenotype towards an activated, proliferative myofibroblast-like phenotype. Activated HSCs synthesize alpha-smooth muscle actin (α-SMA), various cytokines, chemokines, growth factors, and fibroblastic cell markers. In addition, they produce abnormally high levels of ECM proteins and remodeling factors, which eventually results in matrix accumulation [2,3]. However, the signaling pathways that regulate HSC proliferation in liver fibrogenesis remain poorly defined. This makes it difficult to design antifibrotic agents.

Peroxisome proliferator-activated receptors (PPARs) are ligand-inducible transcription factors of the nuclear hormone receptor family, which have been associated with liver fibrosis [4]. Distinct genes produce three PPAR isotypes, PPARα (NR1C1), PPARβ/δ (NR1C2), and PPARγ (NR1C3) [5]. PPARs are activated by a large spectrum of endogenous fatty acids and eicosanoids involved in metabolic and inflammatory pathways [5]. The synthetic PPARα ligand, clofibrate, protects against CCl_4_-induced liver fibrosis [6]. PPARγ, activated by glitazones, down-regulates inflammation, collagen synthesis, HSC activation and proliferation [7,8]. Previous studies have shown that the PPARγ agonist ciglitazone diminished adult liver progenitor (oval) cell response and decreased fibrosis in mice fed with a choline deficient, methionine supplemented diet, while the PPARβ/δ ligand GW501516 did not affect oval cell proliferation or liver fibrosis in the same model [9]. Although PPARβ/δ is highly expressed in HSCs, its function in fibrosis is still debated. In cultured primary rat HSCs, the p38 mitogen-activated protein kinase (MAPK) pathway up-regulated PPARβ/δ expression during the transition into the active phenotype. Furthermore, PPARβ/δ transcriptional activation by the selective synthetic ligand L165041 enhanced proliferation of both quiescent and activated HSCs [10] and PPARβ/δ modulated the expression of vitamin A metabolism-related genes in HSCs undergoing activation [11]. In rats, acute treatment with L165041 and CCl_4_ increased the expression of fibrotic markers [10]. In contrast, PPARβ/δ was protective against azoxymethane and CCl_4_-induced hepatotoxicity and, when activated with the synthetic ligand GW0742, down-regulated proinflammatory gene expression in CCl_4_-treated mice [12,13]. In a model of chronic ethanol-fed rats, L165041 reduced hepatic injury, oxidative stress and DNA damage, and improved the regenerative response in livers [14]. A recent study in mice also demonstrated hepatoprotective and antifibrotic effect of the PPARβ/δ ligand KD3010 in both CCl_4_-induced and cholestatic liver fibrosis, in contrast to GW501516 that had profibrogenic effects [15]. The effect of GW501516 was studied after short exposure (12 days) to CCl_4_ and the molecular mechanism by which GW501516 increased fibrosis was not investigated. These different outcomes suggested that the action of PPARβ/δ might be context-dependent, since the above-mentioned studies included different PPARβ/δ ligands, different rodent species and different models of liver damage. Thus, the role of PPARβ/δ in liver injury remains uncertain, and its signaling pathways for regulating liver fibrosis are unknown.

This study focused on the role of GW501516-activated PPARβ/δ in mouse liver fibrosis after long-term CCl_4_ treatment, which is more relevant to liver diseases in human [2,3]. The results presented below clarified the mechanism by which GW501516-activated PPARβ/δ enhanced HSC proliferation, and may facilitate the development of therapeutic approaches to prevent the progression of liver fibrosis through antagonizing PPARβ/δ.

## Results

### *GW501516-activated PPAR*β*/*δ *increased CCl*_*4*_*-induced hepatic fibrosis*

**Figure 1:**
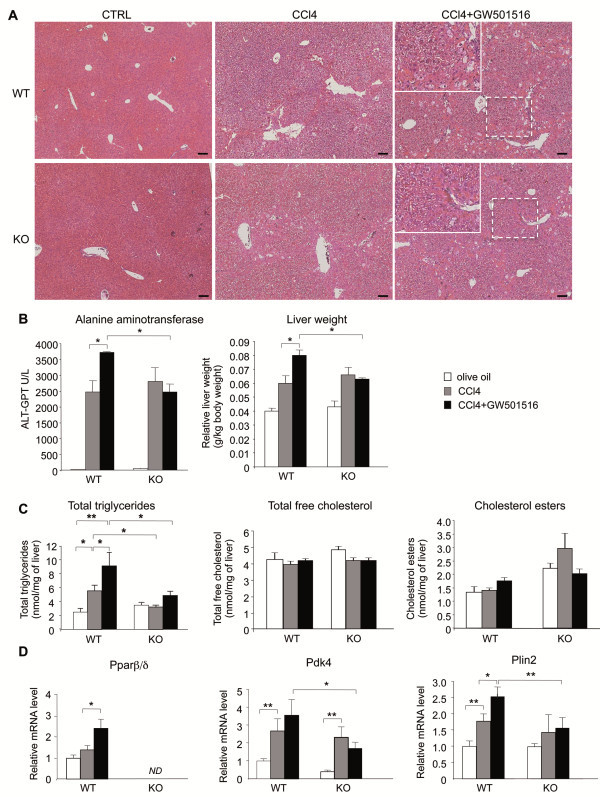
**Effect of GW501516-activated PPARβ/δ on CCl**_**4**_**-induced liver fibrosis and PPARβ/δ target gene expression.A**) Mouse liver sections stained with hematoxylin/eosin after 6 weeks without (CTRL, vehicle) or with CCl_4_ or CCl_4_/GW501516 co-treatment. CTRL: controls treated with 0.5% CMC. Note that olive oil as control gave similar results. WT, wild type mice; KO, PPARβ/δ-null mice. Scale bar: 100 μm. **B**) Serum alanine aminotransferase levels (left) and liver weight (right) at different treatments. **C**) Total hepatic triglycerides (left), total free cholesterol (middle) and cholesterol esters (right) levels with different treatments were determined by gas chromatography. Control mice were treated with olive oil. **D**) qRT-PCR analysis shows expression of (left) *Ppar*β*/*δ and two of its target genes, (middle) *Pdk4* and (right) *Plin2*, in mice treated as indicated. WT, wild type mice; KO, PPARβ/δ-null mice; ND, not detected. Means ± SEM (n=6). * = p<0.05, ** = p<0.01, Student's *t*-test.

To study liver fibrosis, male wild type and PPARβ/δ-null mice received intraperitoneal injections of CCl_4_ twice per week for 6 weeks. The effect of activated PPARβ/δ on liver fibrosis was assessed by treating mice with the well-characterized selective ligand GW501516, in addition to CCl_4_. CCl_4_-treated wild type and PPARβ/δ-null mice developed moderate, centrolobular necrosis with inflammatory, periportal, neutrophil and Kupffer cell/macrophage infiltration. Calcium deposits were found in the necrotic areas (not shown). Liver pathology was slightly more developed in CCl_4_-treated wild type compare to PPARβ/δ-null mice (Figure 1A). This indicated that, in the absence of exogenous activation, PPARβ/δ did only moderately impact liver fibrosis. However, wild type mice co-treated with CCl_4_/GW501516 showed more severe centrolobular necrosis, marked neutrophil infiltration, and degenerated neutrophils and macrophages, including Kupffer cells. This result correlated with serum alanine aminotransferase (ALT) levels and liver weight (Figure 1B). These effects were not observed in similarly treated PPARβ/δ-null mice, which suggested that the GW501516 effect was dependent on PPARβ/δ expression. The expression profiles of *Ppar*α and *Ppar*γ under the different experimental conditions were similar in PPARβ/δ-null mice and wild type mice ( Additional file 1: Figure S1). This indicated that PPARβ/δ deletion probably did not trigger compensatory effects. Nonetheless, it is worth noting that CCl_4_ treatment reduced the expression of *Ppar*α and *Ppar*γ by more than 50%.

Next, because CCl_4_ is known to impact hepatic lipid homeostasis, we tested the distribution of neutral lipids [16]. The total triglycerides increased with CCl_4_, and even more with CCl_4_/GW501516 co-treatment in wild type mice, whereas the different treatments did not significantly affect triglyceride levels in PPARβ/δ-null mice (Figure 1C). The higher levels of triglycerides in the livers of CCl_4_-treated wild type compared PPARβ/δ-null mice may indicate a moderate contribution of PPARβ/δ in the wild type animal, consistent with the liver pathology described above. On the contrary, neither treatment affected the levels of total free cholesterol or cholesterol esters in wild type or PPARβ/δ-null mice. The mechanisms underlying this PPARβ/δ-dependent accumulation of triglycerides, which triggered hepatic steatosis in co-treated wild type mice, is in accordance with marked fibrosis but remain to be explored.

The CCl_4_/GW501516 treatment markedly increased *Ppar*β*/*δ expression in wild type mice. Importantly, the expression of pyruvate dehydrogenase kinase-4 (*Pdk4*) and Perilipin 2 (*Plin2*), two target genes of PPARβ/δ were also increased in wild type mice, which indicated transcriptional activation (Figure 1D). Note that GW501516 alone stimulated the expression of *Pdk4* and *Plin2* in wild type but not PPARβ/δ-null mice (not shown), while CCl_4_ alone also stimulated the expression of *Pdk4* in both wild type and PPARβ/δ-null mice and *Plin2* in wild type mice, but the stimulation was highest in wild type co-treated mice.

Collectively, these results provided evidence that GW501516-dependent PPARβ/δ activity was enhanced in hepatic fibrotic tissues. This suggested that in this model, PPARβ/δ might exacerbate uncontrolled liver repair. This is consistent with the profibrotic effect of GW501516 reported by others, although their studies did not included null mice [15].

### *GW501516-activated PPAR*β*/*δ *increased expression of pro-inflammatory markers and macrophage infiltration in fibrotic livers*

**Figure 2:**
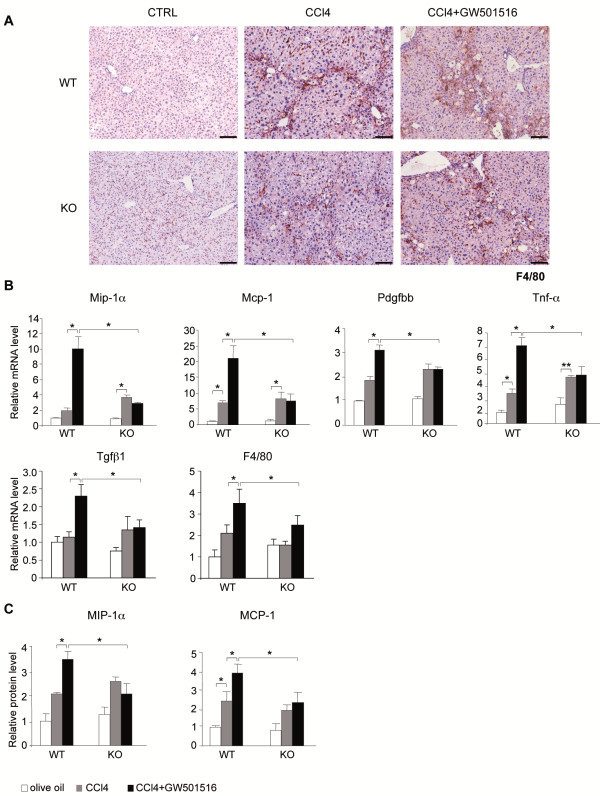
**Ligand-activated PPARβ/δ triggered hepatic macrophage infiltration and inflammatory marker production.A**) Liver sections were immunostained with an antibody against the F4/80 macrophage marker (brown). Nuclei were counterstained with eosin (blue). Liver tissues were obtained from mice treated without (CTRL; 0.5% CMC; note that olive oil as control gave similar results) or with CCl_4_ alone or both CCl_4_/GW501516 for 6 weeks. Eight livers were analyzed for each treatment; one representative example is shown. Scale bar: 100 μm. WT = wild type; KO = PPARβ/δ-null mice. **B**) qRT-PCR analysis shows mRNA expression of *Mip-1*α, *Mcp-1*, *Pdgfbb*, *Tnf-*α*, Tgf*β*1* and *F4/80*. Results are means ± SEM of triplicate assays (n=6). **C**) MIP-1α and MCP-1 protein levels determined from whole cell protein extracts isolated from livers after the indicated treatments. WT = wild type; KO = PPARβ/δ-null mice. Means ± SEM of triplicate assays (n=6). * = p<0.05; Student's *t*-test.

In liver sections from untreated wild type and PPARβ/δ-null mice, F4/80 staining (macrophages, Kupffer cells) was weak (Figure 2A). However, in CCl_4_-injured livers, we measured an important increase in staining, whereas the number and localization of recruited macrophages/Kupffer cells were similar in both genotypes. Most of the damage was located around blood vessels. Administration of GW501516 alone doubled the number of infiltrated macrophages/Kupffer cells in the wild type mice, but not in PPARβ/δ-null mice (not shown).

Consistent with these observations, GW501516/CCl_4_-damaged wild type livers presented high levels of the pro-inflammatory markers such as macrophage inflammatory protein-1α (*Mip-1*α), monocyte chemoattractant protein-1 (*Mcp-1*), platelet-derived growth factor BB (*Pdgfbb*), tumor necrosis factor alpha (*Tnf-*α), transforming growth factor beta 1 (*Tgf*β*1*) and the mouse homolog (*F4/80*) of the EGF-like module-containing mucin-like hormone receptor-like 1 in wild type mice (Figure 2B), which are known to be expressed in activated HSCs, infiltrated Kupffer cells, and other immune cells. Treatment with CCl_4_ alone also induced the mRNA levels of these markers, but to a lower extent and in a PPARβ/δ-independent manner (Figure 2B). This was confirmed at the protein level for MIP-1α and MCP-1 (Figure 2C). Thus, GW501516-activated PPARβ/δ in fibrotic liver increased inflammation, most likely as a consequence of increased immune cell infiltration and HSC activation and proliferation.

### *GW501516-activated PPAR*β*/*δ *increased ECM deposition during fibrosis*

**Figure 3:**
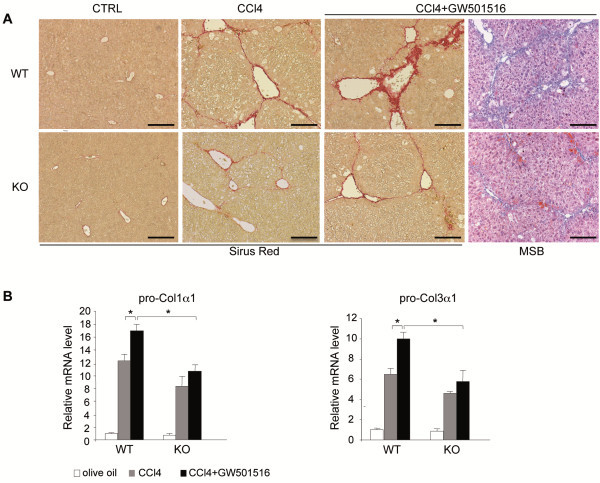
**Ligand activated PPARβ/δ-dependent increase in hepatic pro-fibrogenic marker expression.A**) Sirus red staining of collagen fibers in liver sections from wild type (WT; top row) and PPARβ/δ-null mice (KO; bottom row). Mice were untreated (CTRL; 0.5% CMC; note that olive oil as control gave similar results) or treated with CCl_4_ or CCl_4_/GW501516 for 6 weeks. The far right panels are the samples stained with Martius/Scarlet/Blue to label fibrin in WT and KO mice treated with CCl_4_/GW501516. Sections are representative of six mice in each treatment group. Scale bar: 100 μm. **B**) qRT-PCR analysis shows *pro-Col1*α*1* (left) and *pro-Col3*α*1* (right) mRNA expression after 6 weeks of the indicated treatments in WT and PPARβ/δ KO mice. Results are means ± SD of at least three independent experiments performed in triplicate (* = p<0.05, Student's *t*-test).

Fibrosis is characterized by the deposition of ECM components. They are secreted by activated HSCs during liver repair. Histological sections stained with Sirus red showed normal distributions of collagen around liver blood vessels in untreated wild type and PPARβ/δ-null groups (Figure 3A). CCl_4_ treatment caused a moderate increase in perilobular and centrolobular collagen distributions, widespread pericellular fibrosis, and centro-central fibrotic septa, which was more important in wild type compared to PPARβ/δ-null livers as determined by staining quantification ( Additional file 1: Figure S2). The damages were graded with an Ischak’s score of 2 in most liver sections. Co-administration of GW501516 and CCl_4_ strongly enhanced collagen deposition at the centrolobular and periportal regions, and collagen fibers extended within the lobule and out to the surrounding hepatocytes in wild type mice, but not in similarly-treated PPARβ/δ-null mice. In wild type mice, CCl_4_/GW501516 caused hepatic damage with an Ischak’s score of 3 in most liver sections. This result was supported by quantification of the Sirus Red staining ( Additional file 1: Figure S2) and by staining for fibrin with Martius/Scarlet/Blue (MSB) (Figure 3A right panels). These results showed that GW501516-activated PPARβ/δ in CCl_4_-injured livers enhanced collagen deposition and thus, promoted fibrosis; however, this effect was not observed in PPARβ/δ-null mice.

In line with these observations, CCl_4_ treatment increased *pro-Col1*α*1* and *pro-Col3*α*1* mRNA expression in both wild type and PPARβ/δ-null genotypes, though to a slightly lesser extent in the latter (Figure 3B), in agreement with the Sirus Red staining ( Additional file 1: Figure S2). Interestingly, the combined CCl_4_/GW501516 treatment further induced the expression of these genes compared to CCl_4_ alone only in wild type mice.

Taken together, our observations showed that activation of PPARβ/δ in CCl_4_-injured livers strongly promoted collagen deposition, a hallmark of liver fibrosis.

### *GW501516-activated PPAR*β*/*δ *exacerbated HSC activation and proliferation*

**Figure 4:**
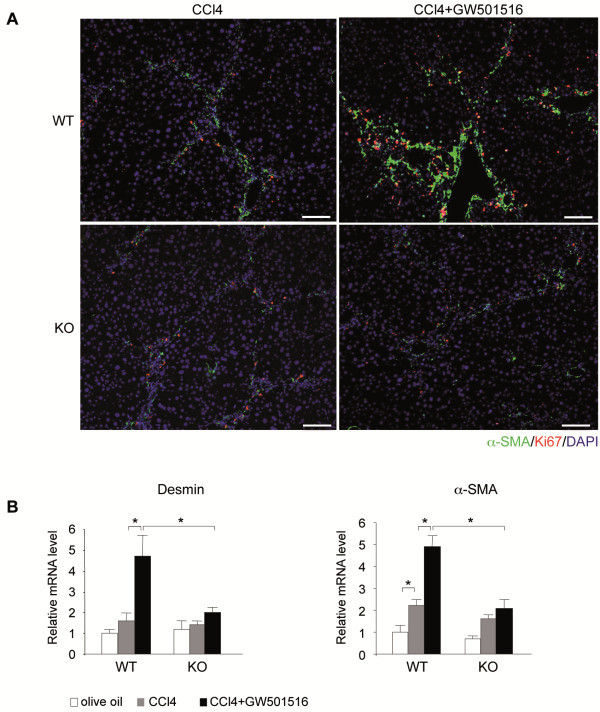
**PPARβ/δ induced HSC proliferation in fibrotic mouse liver.A**) Mouse liver sections were double-immunostained with antibodies against Ki67 (proliferation marker, red) and α-SMA(activated HSC marker, green). Nuclei were stained with DAPI (blue). Mice were treated with CCl_4_ or CCl_4_/GW501516 for 6 weeks. Sections are representative of 6 mice in each treatment group. Scale bar: 100 μm. **B**) qRT-PCR analysis shows Desmin and α*-SMA* mRNA expression after 6 weeks of the indicated treatments in wild type and PPARβ/δ KO mice. Results are means ± SEM of triplicate experiments (n=6). * = p<0.05, Student's *t*-test.

During fibrogenesis, HSCs proliferate and transdifferentiate to myofibroblasts that express α-SMA [1]. Immunohistochemistry with α-SMA and Ki67 antibodies showed a quasi absence of staining in untreated wild type and PPARβ/δ-null liver sections (not shown). After chronic CCl_4_ exposure, many α-SMA and Ki67 positive cells were observed within the lobule and in the fibrotic septa, at slightly higher levels in wild type compared to PPARβ/δ-null mice (Figure 4A). These results indicated that CCl_4_ treatment induced the activation and proliferation of HSCs. Administration of GW501516 to CCl_4_-injured livers further increased the lobular distribution and the number of activated and proliferative HSCs only in wild type mice. Since GW501516 alone had no effect on HSC proliferation in absence of CCl_4_ treatment in wild type mice (not shown), this implied that CCl_4_ activation of the HSCs was a prerequisite for the PPARβ/δ-dependent effect on cell proliferation.

Consistent with these results, CCl_4_ administration increased the levels of α*-Sma* mRNA by 2-fold in both wild type and PPARβ/δ-null mice (Figure 4B). Combined administration of GW501516 and CCl_4_ strongly increased the expression of both *Desmin* and α*-Sma* transcripts in wild type mice, but not in PPARβ/δ-null mice. This suggested that the agonist action was PPARβ/δ-dependent. These results demonstrated that ligand-activated PPARβ/δ increased the proliferation of activated HSCs in CCl_4_-injured mouse liver, a cellular process that promotes and amplifies fibrosis.

### *PPAR*β*/*δ *increased LX-2 cell proliferation through p38 and SAPK/JNK MAPKs via upstream PI3K activation*

**Figure 5:**
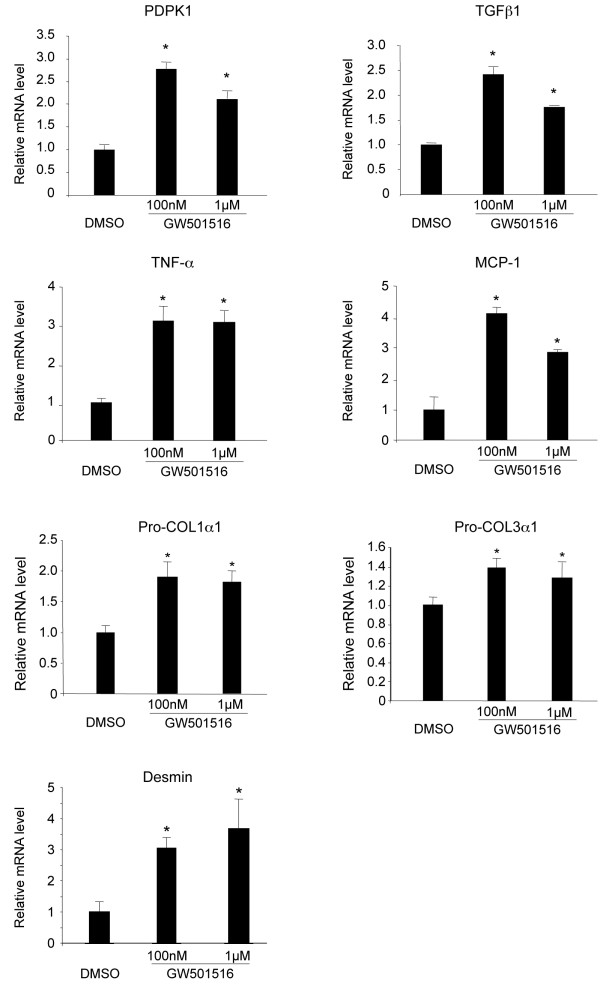
**Expression of PPARβ/δ and PPARβ/δ-dependent genes in LX-2 human hepatic stellate cells.** Human LX-2 stellate cells were serum-starved for 24 h, and then treated with DMSO (0.01%), 100nM GW501516, or 1μM GW501516 for 48 h. qRT-PCR analysis shows mRNA levels of *PDPK1, TGF*β*1, TNF-*α*, MCP-1, Pro-COL1*α*1, Pro-COL3*α*1* and *Desmin*. Results are means ± SD of at least three independent experiments performed in triplicate. * = p<0.05, Student's *t*-test.

**Figure 6:**
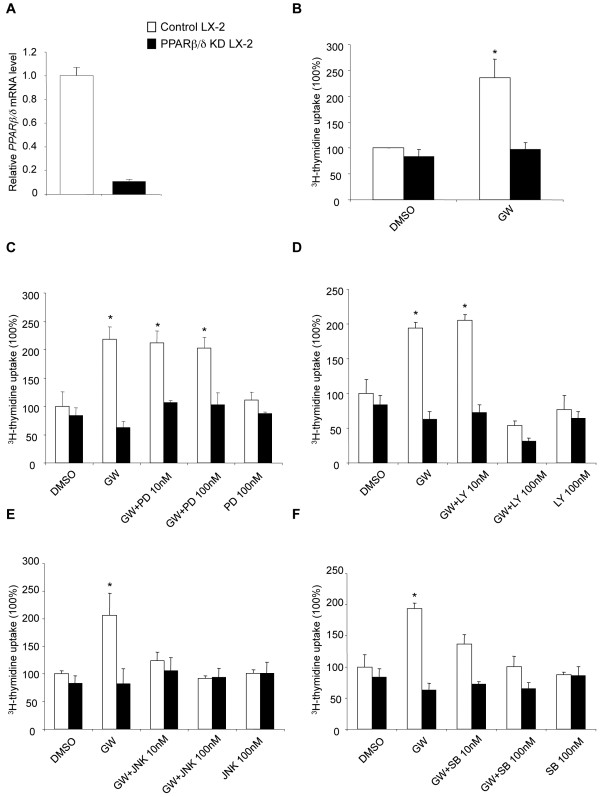
**PPARβ/δ controls pathways that regulate proliferation of LX-2 cells.** Stable PPARβ/δ knockdown (KD) was performed by lentiviral transduction of LX-2 cells with a siRNA against *PPAR*β*/*δ. Control LX-2 cells were transduced with the control vector. **A**) qRT-PCR showed reduced *PPAR*β*/*δ expression in PPARβ/δ KD LX-2 cells. The control values were set to 1. Results are means ± SD of at least three independent experiments performed in triplicate. **B-F**) Control and PPARβ/δ KD LX-2 cells were starved for 24 h in serum-free media and then treated for 48 h with DMSO (control) or (**B**) 100 nM GW501516, or (**C-F**) 100 nM GW501516 in the presence or absence of 10 nM or 100 nM of (**C**) PD98059, (**D**) LY294002, (**E**) JNK inhibitor II, or (**F**) SB202190. All inhibitors were added 30 min before GW501516 treatment. Cell proliferation was determined by [^3^H]-thymidine incorporation. Values are expressed as a percentage of the values from DMSO-treated LX-2 cells, which were set to 100%. Values represent means ± SEM from at least five independent experiments performed in triplicate; * = p<0.05 compared to DMSO-treated cells.

The molecular mechanisms underlying PPARβ/δ regulation of activated HSC proliferation after liver injury are not known. To address this question, we first explored whether treatment with GW501516 also impacted gene expression in human activated HSC LX-2 cells, which express key genes for hepatic fibrosis and are phenotypically similar to primary activated human HSCs *in vivo*[17]. These cells are in a pre-activated state and they progressively express activation markers after cultivation [18]. Therefore, they present some similarity with CCl_4_ activated HSCs. The results presented in Figure 5 showed that the expression of genes stimulated in the mouse liver after CCl_4_/GW501516 treatment (see Figure 1,4) was also enhanced by GW501516 in human HSC LX-2 cells. This observation prompted us to use these cells to identify the signaling pathways involved in HSC proliferation. For this purpose, we stably knocked down (KD) PPARβ/δ in human LX-2 stellate cells with lentiviral constructs that contained short interfering RNAs (siRNAs) against *PPAR*β*/*δ mRNA. This resulted in a 90% reduction in *PPAR*β*/*δ mRNA expression (Figure 6A).

The proliferation of LX-2 cells was measured in a [^3^H-methyl]-thymidine incorporation assay. Treatment with GW501516 increased proliferation by 2.5 fold compared to control DMSO-treated cells. This effect was blunted in PPARβ/δ KD LX-2 cells (Figure 6B). To elucidate the cascade of events between activated PPARβ/δ and increased LX-2 cell proliferation, we specifically inhibited several signaling pathways that might be involved in activated HSC proliferation. The MAPK extracellular signal-regulated kinase 1/2 (Erk1/2) pathway inhibitor, PD98049 (MEK1 inhibitor), had no effect on GW501516-inducible LX-2 cell proliferation (Figure 6C). In contrast, pre-incubation with the PI3K pathway inhibitor, LY294002, followed by exposure to GW501516 for 48 h inhibited PPARβ/δ-dependent LX-2 cell proliferation at the dose of 100 nM (Figure 6D). This implicated a PI3K-dependent pathway in GW501516-induced HSC proliferation. Next, we applied inhibitors of two PI3K downstream targets, the stress-activated protein kinase/c-Jun NH2-terminal kinase (SAPK/JNK) and p38 MAPK. These inhibitors (JNK inhibitor II and SB202190, respectively) also abolished activated PPARβ/δ-dependent stimulation of LX-2 cell proliferation (Figure 6E and 6F). This result identified two main signaling pathways, SAPK/JNK and p38 MAPK, which are involved in PPARβ/δ-induced HSC proliferation.

### *PPAR*β*/*δ *increased phosphorylation of the PI3K-dependent PKC*α*/*β*II/MLK3 signaling pathway, which leads to p38 and JNK MAPKs activation*

**Figure 7:**
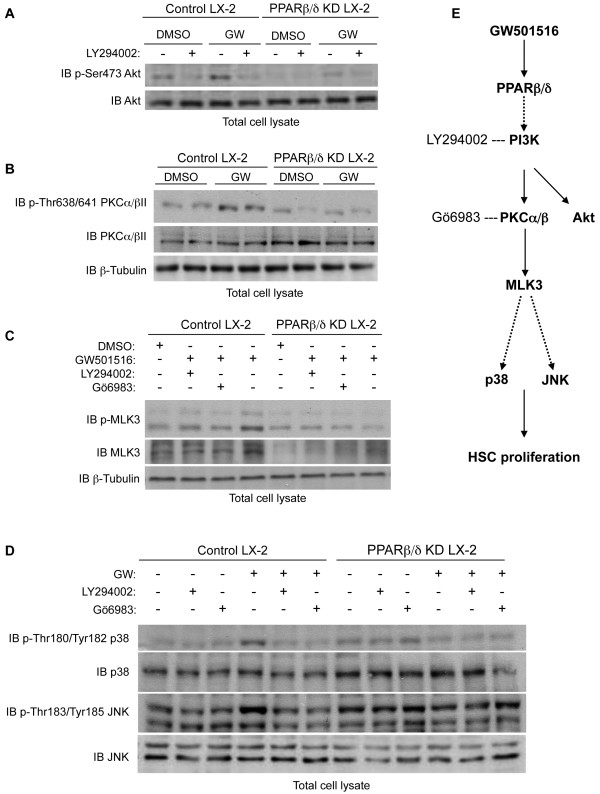
***PPARβ/δ*induced a signaling pathway involving* PI3K/PKCα/βII/MLK3/p38*and*JNK MAPKs.*** Control and PPARβ/δ KD LX-2 cells were serum-starved for 24 h, and then pre-treated with the indicated inhibitor for 30 min before incubation with 100 nM GW501516 or 0.01% DMSO. After total cell lysis, proteins were resolved by immunoblot (IB).β-Tubulin served as the loading control. **A**) IB shows phosphorylation of Akt on Ser473 in the presence or absence of PI3K inhibitor LY294002 (20 μM). **B**) IB shows PPARβ/δ-dependent PKCα/βII protein expression and phosphorylation. **C**) IB shows MLK3 protein expression and phosphorylation with or without PI3K inhibitor LY294002 (20 μM) or PKC inhibitor Gö6983 (7 μM). **D**) IB shows p38 and JNK protein expression and phosphorylation in the presence or absence of LY294002 (20 μM) or Gö6983 (7 μM). IBs are representative of three independent experiments. **E**) Schematic model for the regulation of human hepatic LX-2 stellate cell proliferation by GW501516-activated PPARβ/δ. Ligand activation of PPARβ/δ enhances PI3K activity, resulting in activation of PKCα/βII and downstream MLK3. MLK3 signaling eventually results in increased phosphorylation of p38 and JNK MAPKs, which are known to enhance HSC proliferation.

Because the PI3K pathway appeared to be required for PPARβ/δ-induced LX-2 cell proliferation, we analyzed the phosphorylation level of the PI3K downstream target Akt, a well-validated marker for PI3K activity. We observed a PPARβ/δ-dependent increase in Akt phosphorylation on serine 473 in control LX-2 cells (Figure 7A). This effect was inhibited by the PI3K inhibitor LY294002 and was blunted in PPARβ/δ KD LX-2 cells. Moreover, the Akt protein expression level was not modified in control or PPARβ/δ KD LX-2 cells. This indicated that Akt phosphorylation was both PPARβ/δ- and PI3K-dependent.

It is known that PI3K phosphorylation and stimulation of PKC are among the earliest events in the activation of MLK3, a MAPK kinase kinase (MAPKKK). MLK3 stimulates the MAPKKs MKK3/6 and MKK4, which finally activate p38 and JNK MAPKs in the last steps of initiating HSC proliferation [19-22]. Among the different PKC isoforms tested in control LX-2 cells, GW501516 induced only the phosphorylation of PKCα/βII on Thr638/641. This phosphorylation was not observed in similarly treated PPARβ/δKD LX-2 cells (Figure 7B). In addition, GW501516-activated PPARβ/δ had no effect on PKCα/βII protein expression levels. Interestingly, GW501516 increased both MLK3 protein expression and phosphorylation specifically in control LX-2 cells (Figure 7C). This effect was blunted by inhibitors of PI3K (LY294002) and PKC (Gö6983). In line with these results, GW501516 treatment in control LX-2 cells induced a PPARβ/δ-dependent phosphorylation of p38 at Thr180/Tyr182 and JNK at Tyr183/Thr185 (Figure 7D). This effect was also dependent on PI3K and PKC activation, as shown by LY294002 and Gö6983 treatments, which abolished the GW501516-induced phosphorylation of p38 and JNK (Figure 7D).

Collectively, these results were consistent with our data on HSC proliferation (Figure 6), and suggested that GW501516 stimulated HSC proliferation by activating p38 and JNK MAPKs, *via* an upstream signaling pathway involving PI3K, PKCα/βII and MLK3 (Figure 7E).

### *Putative relevance of PPAR*β*/*δ *in human liver fibrosis*

**Figure 8:**
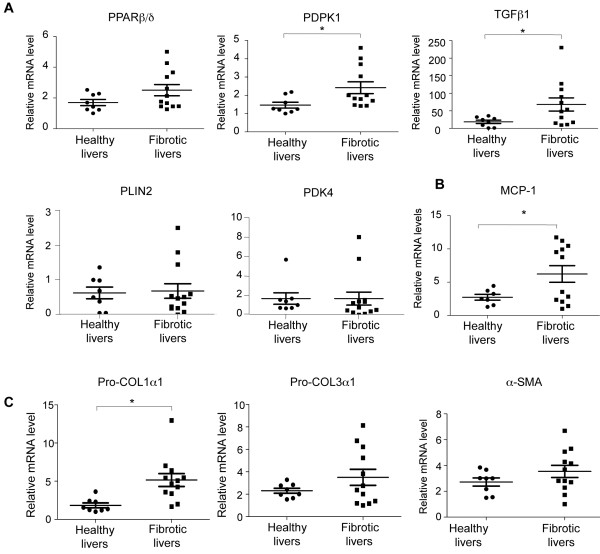
**Expression of *PPARβ/δ, PPARβ/δ *target genes, inflammatory and fibrosis markers in human fibrotic livers.** mRNA expression in biopsies from healthy (n=8) and fibrotic (n=12) human livers is shown. qRT-PCR analysis shows *PPAR*β*/*δ mRNA expression, and mRNA expression of the PPARβ/δ target genes *PDPK1, TGF*β*1, PLIN2 and PDK4* (**A**), the inflammatory marker *MCP-1* (**B**), the fibrosis markers *pro-COL1*α*1* and *pro-COL3*α*1* and the HSC marker α*-SMA* (**C**) * = p<0.05, Student's *t*-test.

To test whether the activity of PPARβ/δ may also be relevant to the development of human liver fibrosis, its levels were measured in healthy subjects and patients with alcoholic fibrosis/cirrhosis. In diseased livers, there was a clear trend towards higher *PPAR*β*/*δ expression, and more heterogeneous expression was observed among fibrotic livers compared to healthy livers (Figure 8A). This heterogeneity was observed for all the mRNAs tested and may reflect differences in the severity of fibrosis between diseased individuals. Importantly, two well-established PPARβ/δ target genes, phosphoinositide dependent kinase 1 (*PDPK1*) and transforming growth factor beta-1 (*TGF*β*1*), showed increased expression in diseased livers, which may reflect higher PPARβ/δ transcriptional activity (Figure 8A). However, the expression of *PLIN2* and *PDK4* was not increased (Figure 8A). Furthermore, the expression of inflammatory (*MCP-1;* Figure 8B) and fibrosis (*pro-COL1*α*1* Figure 8C) marker genes was significantly increased in the biopsy samples, in agreement with results obtained in mouse liver. The expression of *pro-COL3*α*1* and α*-SMA* showed a similar trend, albeit without reaching statistical significance (Figure 8C). Together, these results obtained from human subjects suggest that a similar mechanism of fibrosis development also exists in man, but a direct mechanistic implication of PPARβ/δ in this species remains to be substantiated.

## Discussion/conclusion

This study identified GW501516-activated PPARβ/δ as a promoter of uncontrolled liver repair, which results in fibrosis, most likely *via* p38- and JNK-dependent stimulation of HSC proliferation. This healing function of PPARβ/δ is reminiscent of its role in skin wound healing [23]. Fibrosis is a response to a variety of chronic damaging stimuli. It can cause an alteration in liver structure that may lead to excessive deposition of ECM, apoptosis of hepatocytes and inflammation [2,3]. During the fibrogenic response, activated HSCs proliferate and indeed produce an excess of ECM and pro-inflammatory proteins.

To date, the role of PPARβ/δ has been unclear in this hepatic repair process, which often degenerates to liver disease. In the present study, mice were exposed to both CCl_4_ and a PPARβ/δ selective ligand for a long period of time (i.e., 6 weeks). We found that agonist-activated PPARβ/δ had an additive or synergistic effect with CCl_4_ on the production of inflammatory cytokines, pro-fibrotic ECM proteins and HSC markers, and on the accumulation of hepatic triglycerides. In line with our results, a recent study also demonstrated a profibrotic effect of the PPARβ/δ ligand GW501516 after short-term CCl_4_ administration in mice [15]. In contrast to our data, this study did not identify the molecular mechanisms by which GW501516-activated PPARβ/δ induced the fibrotic process. Furthermore, this profibrotic action of activated PPARβ/δ was consistent with our previous study in a rat model of acute CCl_4_-induced liver injury treated with a different PPARβ/δ ligand (L165041). In this model, we found increased expression of *Col1Î±1, Î±-SMA,* and lysyl oxidase with CCl_4_/L165041 treatment [10]. In contrast, other studies concluded that GW0742- or KD3010-activated PPARβ/δ attenuated CCl_4_-induced hepatotoxicity [13,15]. Our present findings suggest that CCl_4_ treatment alone causes only a weak activation of PPARβ/δ. For example, we showed that CCl_4_ did not result in important differences in the expression of several genes when wild type and PPARβ/δ-null mice were compared, but GW501516/CCl_4_ co-treatment strongly induced these genes only in wild type mice. Importantly, we found that several genes that were strongly stimulated by the combined action of CCl_4_ and GW501516 were also expressed at higher levels in hepatic tissue of patients with confirmed alcohol-induced liver fibrosis/cirrhosis. The discrepancies among different studies may be due to differences in the ligands used, the dose applied, and duration of administration. For instance, different ligands may present different pharmacophore features resulting in different physiological outcomes. In future studies, it will be interesting to use cell type-specific deletion of PPARβ/δ *in vivo* to evaluate the individual contribution of stellate cells, macrophages/Kupffer cells and hepatocytes to the observed PPARβ/δ-dependent profibrotic or protective effects.

The underlying mechanism of the PPAR-dependent stimulation of HSC proliferation *in vivo* was unveiled in the human LX-2 HSC line. These cells express key genes involved in hepatic fibrosis [17]. Addition of the GW501516 ligand activated PPARβ/δ in these cells and increased proliferation after 48 h, whereas no change in proliferation was observed in the PPARβ/δ KD cells. Similar to the *in vivo* results, the PPARβ/δ ligand also increased the expression of pro-inflammatory and profibrotic factors. These results were consistent with our previous study, which showed that L165041-induced activation of PPARβ/δ in cultured activated primary HSCs enhanced proliferation and profibrotic factor expression [10].

In the present study, we also investigated genes that were not direct targets of PPARβ/δ, but rather reflected the activation of PPARβ/δ-dependent signaling pathways. We found that PPARβ/δ regulated the PI3K, p38 MAPK, and SAPK/JNK pathway, which is known to be involved in cell proliferation. We also found that Erk1/2 MAPK and nuclear factor-κB (NF-κB) signaling did not contribute to PPARβ/δ-induced HSC proliferation (data not shown). In fact, it was previously shown that MAPKs p38 and JNK were positive regulators of HSC proliferation [1,24,25]. Those studies showed that multiple stress stimuli increased the activity of SAPK/JNK and p38 MAPKs, which in turn activated several transcription factors implicated in cell proliferation and differentiation [26]. The present study revealed the novel finding that, during fibrosis, these factors were regulated by GW501516-activated PPARβ/δ. Thus, it was of interest to unveil how PPARβ/δ controlled this paramount signaling pathway.

Our results showed that GW501516-activated PPARβ/δ enhanced phosphorylation of p38 and SAPK/JNK MAPKs without changing their expression levels. This suggested that PPARβ/δ was involved in the transcriptional regulation of upstream kinases. In fact, PPARβ/δ-dependent phosphorylation of p38 and JNK was suppressed by inhibitors of PI3K (LY294002) and PKC (Gö6983). In addition, we observed a PPARβ/δ-dependent phosphorylation of Akt at Ser473. Consistent with this finding, ligand-activated PPARβ/δ in skin increased keratinocyte survival upon exposure to stress through PI3K signaling; this was reflected by increased Akt1 activity [27]. Interestingly, PKCs are downstream targets of activated PI3K. It was previously demonstrated that acetaldehyde induced PKC activation, which then increased HSC proliferation and activation [28-31] and collagen production [32,33]. Thus, we hypothesized that, in HSCs, PPARβ/δ might also upregulate PDPK1 (*Pdpk1*) and downregulate phosphatase and tensin homolog (*Pten*) expression. This would activate, *via* PI3K and PKC, the Ser/Thr protein kinase MLK3, a cytokine-activated MAP3K known to regulate JNK, p38, and Erk1/2 [21,34]. We showed for the first time that GW501516 increased MLK3 protein expression and phosphorylation in a PPARβ/δ-dependent manner; furthermore, PKC inhibitors blocked MLK3 activation. Thus, GW501516 indirectly activated MLK3, a downstream target of PKC. It was previously established that MLK3 phosphorylates and activates the MAP2K isoforms MKK4/7 and MKK3/6, which then activate JNK and p38, respectively [19,20,34,35]. Interestingly, because GW501516 increased both the phosphorylation and expression levels of MLK3 (Figure 7C), MLK3 may be both a direct and indirect target of activated PPARβ/δ. Recent studies demonstrated that RNAi-mediated knockdown of MLK3 inhibited serum-stimulated cell proliferation, tumor cell proliferation, and growth factor/cytokine-induced JNK, p38, and Erk1/2 activation [21,22]. These cells also exhibited destabilized B-Raf/Raf1 complexes [36]. Furthermore, CEP-1347, the small-molecule inhibitor of all MLK members, caused reductions in pulmonary fibrosis [37], pancreatitis [38], and neurodegeneration [39] by inhibiting JNK activation.

In conclusion, this report is the first to show that GW501516-activated PPARβ/δ could enhance both the p38 and JNK MAPKs signaling pathways, and thus, increase HSC proliferation in liver injuries. Furthermore, we showed that PPARβ/δ activated p38 and JNK by phosphorylating PI3K/PKC/MLK3 components (Figure 7E). We propose that activated PPARβ/δ increased HSC proliferation, which then exacerbated inflammatory and fibrotic processes during liver injuries. Taken together, these findings showed that GW501516-activated PPARβ/δ represents an important regulatory step in HSC proliferation. Finally, the role of PPARβ/δ and its activation in HSC proliferation in liver fibrosis should be considered when evaluating PPARβ/δ agonists as potential therapeutic agents for broad applications; for example, a phase II clinical trial is currently testing GW501516 as a treatment for dyslipidemia. Furthermore, it will be important, in the future, to evaluate whether natural ligands can achieve effects similar to those of GW501516.

## Materials and methods

### Reagents

CCl_4_ was obtained from VWR International and olive oil was from Sigma Aldrich. GW501516 was synthesized by Kissei Pharmaceutical Co. Ltd. (Matshumoto, Japan).

### CCl_4_ treatment of mice

Wild type and PPARβ/δ-null [23] 6–8 week-old male mice of a mixed genetic background Sv129/C57BL/6, were maintained at 23 °C on a 12-h light–dark cycle with free access to water and a standard diet. To induce liver fibrosis, 6 wild type and 6 PPARβ/δ-null mice received repeated intraperitoneal injections (1 μl/g body weight) of CCl_4_:olive oil (1:1) twice per week for 6 weeks. Injection of olive oil alone served as a control. In addition to the CCl_4_-treatment, 6 wild type and 6 PPARβ/δ-null male mice received 10 μg/kg/day of GW501516 in 0.5% carboxymethyl cellulose (CMC), or GW501516 and CMC alone by gavage once per day for 6 weeks. At the end of the experimental period, blood samples were collected by retro-orbital puncture for measurement of the liver damage specific enzyme alanine transaminase (ALT) and neutral lipid analysis and the mice were then killed by cervical dislocation. After weighing, livers were either rapidly frozen in liquid nitrogen for later analyses or immediately prepared for immunocytochemistry studies and pathological examinations. All treatments were repeated in 3 independent experiments (n=6/genotype). All experiments involving animals were approved by the Veterinary Office of the Canton Vaud (Switzerland) in accordance with the Federal Swiss Veterinary Office Guidelines and conformed to the European Commission Directive 86/609/EEC and the “Guide for the Care and Use of Laboratory Animals” (NIH publication 86–23 revised 1985).

### Neutral lipid analysis

Hepatic lipids were determined by gas chromatography [40].

### Patients and biopsies

Liver biopsies were collected by transparietal puncture from 8 healthy individuals and 12 patients (10 males, 2 females; aged 48–69 years) with alcohol-caused liver fibrosis or cirrhosis, diagnosed on clinical, biological, and histological grounds [41]. Total RNA was isolated from the liver biopsies with TRIzol reagent (Invitrogen, Carlsbad, CA) and gene transcription was analyzed by quantitative reverse-transcription PCR. All clinical investigations were conducted according to the principles expressed in the Declaration of Helsinki.

### Total RNA isolation, reverse transcription PCR (RT-PCR), and qRT-PCR

Total RNA was extracted from frozen mouse liver samples, from human liver biopsies, or from LX-2 cells with TRIzol reagent (Invitrogen), according to the manufacturer’s instructions. Single-stranded cDNA templates were generated by reverse transcription with Superscript II reverse transcriptase (Invitrogen). For qRT-PCR (TAQ MAN), the cDNA equivalent of 10 ng of total RNA was amplified. All primers, including those specific for amplifying mRNA of mouse or human *PPARβ/δ, PPARα , PPARγ TNF-α, MCP-1, MIP-1α, TGF-β1, PDGFBB, pro-Col1α1, pro-Col3α1, α-SMA, Desmin, PDPK1*, and *PDK4*, were purchased from Applied Biosystems. Fluorescence was quantified with an ABI Prism 7900HT SDS system (Applied Biosystems). The following housekeeping genes were used to normalize mouse liver samples: *Eef1A1*, *mRps9*, *mGapdh*; human biopsies: *RPS18*, *hTBP*; and LX-2 cells: *hGUSB*. Relative mRNA expression levels were calculated with the comparative Ct method (User Bulletin # 2, Applied Biosystems) and qBase software. All values represent means from treated samples compared to means from control samples (vehicle- or olive oil-treated wild type mice or DMSO-treated LX-2 cells), which were set to 1.

### Histological analysis and Sirus Red staining

Liver specimens were fixed in 4% paraformaldehyde (PAF) for 24 h, and then embedded in paraffin. Tissues sections (4 μm) were stained with hematoxylin/eosin (H/E), for routine examination, or with Sirus Red for Col1α1 and Col3α1 visualization.

### Liver pathology

To determine the degree of necroinflammatory liver injury, mouse liver sections were submitted to blind histopathologic examinations (grading according to Ischak’s score) by an independent pathologist (see Acknowledgements).

### LX-2 cells stimulation by GW501516 and Western blot analysis

When indicated, control and LX-2 cells treated with siRNA against PPARβ/δ were first pre-incubated for 30 min in serum-free media with 20μM LY294002 (PI3K inhibitor) or 7 nM Gö6983 (PKC inhibitor, Calbiochem) before the addition of GW501516 for 24 h. Total cell proteins were extracted in ice-cold lysis buffer (10 mM Tris–HCl pH 7.5, 150 mM NaCl, 5 mM NaF, 1% Triton X-100, 0.1% SDS) supplemented with the following protease inhibitors: 2 μg/ml Aprotinin, 1 μg/ml Leupeptin, 2 μg/ml Pepstatin A, 1 mM phenylmethylsulfonyl fluoride, 1% deoxycholic acid, and 1 mM Na_3_VO_4._ After quantification, 30 μg of proteins were separated by SDS-PAGE and subjected to immunoblotting. All primary antibodies were diluted at 1/1000 and incubated overnight in 1× TBS 0.1% Tween-20, 5% nonfat milk. Anti-phospho Akt (Ser473), anti-phospho PKCα/βII (Thr638/641), anti-phospho MLK3 (Thr277/Ser281), anti-phospho p38 MAPK (Thr180/Tyr182), anti-phospho JNK/SAPK (Tyr183/Tyr185), anti-Akt, anti-PKCα, anti-MLK3, anti-p38 MAPK, and anti-SAPK/JNK were from Cell Signaling; anti-β-tubulin (loading control) was from BD Pharmingen^TM^. The signals were detected with an ECL detection kit (Amersham Pharmacia Biotech), according to the manufacturer's instructions. The ScanImage densitometry program was used for quantification, and signals were normalized to the β-tubulin signal.

### HSC proliferation assay

Control LX-2 and PPARβ/δ KD LX-2 cells were plated in 24-well culture plates at a seeding density of 3×10^4^ and incubated in DMEM with 2% FCS. One day later, they received serum-free medium for 24 h. Thereafter, they were treated with 0.01% DMSO (control), or 100 nM GW501516 for 48 h in serum-free medium. Alternatively, cells were pre-incubated for 30 min with 10 nM or 100 nM of SB202190 (p38 MAPK inhibitor), JNK inhibitor II, LY294002 (PI3K inhibitor), or PD98059 (MEK1 inhibitor) before the addition of 100 nM GW501516 for 48 h in serum-free medium. During the last 13 h of these treatments, 1 μCi/well of [^3^H-methyl]-thymidine was added. Then, the culture media were discarded, plates were placed on ice, cells were washed with ice-cold PBS, and then fixed in 500 μl ice-cold 10% trichloroacetic acid (TCA) for 20 min. TCA was removed, and 100 μl cell dissociation solution (0.2M NaOH, 1% SDS) was added to each well. Cells were incubated with gentle shaking for 10 min at room temperature. The samples were neutralized with 100 μl of 0.2 M HCl and transferred to vials with 3 ml scintillation cocktail. The scintillation counts (cpm) for treated control LX-2 cells and PPARβ/δ KD LX-2 cells were expressed as a percentage of the counts measured for the DMSO treated LX-2 cells, which were set to 100%.

### Measurements of hepatic MCP-1 and MIP-1 protein expression

Simultaneous quantifications of MCP-1 and MIP-1*a* levels in the livers of treated wild type and PPARβ/δ-null mice were performed with the mouse cytokine/chemokine LINCOplex KIT, 96-well plate assay (LINKO Research, MCYTO-70K) on a Luminex^R^100. Similar sized pieces of frozen liver samples were homogenized with a power homogenizer (Polytron) in 1 ml of ice-cold PBS with the complete protease inhibitor cocktail. Lysates were incubated on ice for 10 min, followed by centrifugation at 13,000 rpm for 20 min at 4 °C. Supernatants were collected, and centrifugation was repeated several times, until the sample was clear of debris. The isolated proteins were quantified with the Bradford assay (BioRad). An immunoassay was run with 25 μl protein lysate to determine the cytokine/chemokine levels (pg/ml) in the liver according to the manufacturer’s instructions. All data were normalized to the protein concentration, and values were expressed relative to the value measured in vehicle-treated (olive oil) wild type mice, which was set to 1 (n=6 for each treated group).

### Immunohistochemistry

To detect proliferating and activated HSCs, paraffin-embedded liver sections (4 μm) were double immunostained with the anti-αSMA antibody, as a marker for activated hepatic stellate cells, and anti-Ki67, as a marker for cell proliferation. Briefly, after deparaffinization, the antigen unmasking step was performed in 0.01 M citrate buffer, pH 6.0, by heating sections to 100 °C in a microwave oven for 20 min. After washing, sections were blocked in 1% BSA/1×PBS for 30 min at room temperature, and then incubated overnight at 4 °C with mouse anti-α-SMA (1:50) and rabbit anti-Ki67 (1:100) or with anti-F4/80 (1:10) antibody in blocking buffer. After washing, slides were incubated with the appropriate secondary antibodies for 40 min in blocking buffer. Secondary antibodies were anti-mouse IgG Cy3 (1:100) and anti-rabbit IgG FITC (1:400) for anti-α SMA/anti-Ki67, or anti-rat A568 (1:200) for anti-F4/80. After washing 3 times, slides were incubated for 5 min in DAPI for nuclear staining. Then, the sections were rinsed in water and mounted with DABCO for confocal-microscopy.

### *Knockdown of PPAR*β*/*δ *in LX-2 cells by lentiviral infection*

The following sequence was chosen to target the mouse PPARβ/δ sequence: 5'-GCACATCTACAACGCCTAC-3'. This sequence was 100% identical to the human PPARβ/δ sequence. A BLAST search ensured that the sequences would not target other RNAs in a nonspecific manner. The short interfering RNA (siRNA) was cloned into a pLV-TH lentivirus vector under the control of the polymerase III-dependent H1 promoter [42]. In addition, an internal cassette allowed expression of the green fluorescent protein (GFP) marker gene under the control of the elongation factor (EF-1) α promoter [43]. In our study, the empty pLV-TH vector, which contained all the features, but not the siRNA, was designated the control, and the pLV-TH vector containing PPARβ/δ siRNA was designated psiPPARβ/δ. All recombinant lentiviruses were produced by transient transfection of 293T cells according to standard protocols. Briefly, subconfluent 293T cells were co-transfected with 20 μg of the control vector, pLV-TH, or the PPARβ/δ-targeted vector, psiPPARβ/δ, 15 μg of pCMV-∆R8.91, and 5 μg of pMD2G-VSVG by calcium phosphate precipitation. The medium was changed after 16 h, and the recombinant lentiviruses were harvested 24 h later. The lentiviral infection efficiency in LX2 cells was monitored by the percentage of GFP-expressing cells detected by FACS analysis. At an infection multiplicity of 60, 90% of the LX-2 cells expressed GFP 48 h after transduction. The infected cells were then harvested, and total RNA was extracted. A qRT-PCR analysis demonstrated that PPARβ/δ was knocked down (KD) by 90% in the PPARβ/δ KD LX-2 cells compared to control-infected LX-2 cells (Figure 7A).

### Statistical analysis

Data are expressed as means ± SEM or SD for treated wild type and PPARβ/δ-null mice (n=6), or as the means ± SEM or SD of several independent experiments performed in triplicate for LX-2 cells. Statistical significance was determined with the Student's *t*-test.

## Abbreviations

HSCs: Hepatic stellate cells; ECM: Extracellular matrix; PPAR: Peroxisome proliferator-activated receptor; CCl_4_: Carbon tetrachloride; α-SMA: Alpha-smooth muscle actin; CMC: Carboxymethyl cellulose; ALT: Alanine transaminase; PDPK1: Phosphoinositide dependent kinase 1; PDK4: Pyruvate dehydrogenase kinase 4; PLIN2: Perilipin 2; TGF-β: Transforming growth factor-beta; MIP-1α: Macrophage inflammatory protein-1α; MCP-1: Monocyte chemoattractant protein-1; PDGFBB: Platelet-derived growth factor BB; TNF-α: Tumor necrosis factor alpha; pro-Col1α1: Pro-collagen type I α1; pro-Col3α1: Pro-collagen type III α1; KD: Knocked down; Akt: Protein kinase B; MLK3: Mixed-lineage protein kinase 3.

## Competing interests

The authors declare that they have no competing interests.

## Authors’ contribution

RK, AM, PD, and WW designed the study and experiments. RK, AM, EG, FS, HG, DD and PD carried out experiments and interpreted the findings. WW supervised the study. RK, AM and WW prepared the manuscript, which was revised and approved by all authors.

## Supplementary Material

Additional file 1 **Supporting Figure 1. ***Effect of CCl*_*4 *_*and CCl*_*4 *_*/GW501516 treatment on Ppar*α *and Pparγ expression in mouse liver.***Supporting Figure 2.***Quantification of Sirus Red staining in mouse liver sections.* (PDF 143 kb) Click here for file
