# Supplementary material for: GW501516-activated PPARβ/δ promotes liver fibrosis via p38-JNK MAPK-induced hepatic stellate cell proliferation
Source: Cell Biosci. 2012 Oct 10;2:34. doi: 10.1186/2045-3701-2-34 (PMC3519722; doi:10.1186/2045-3701-2-34)
Supplement: Additional file 1 — Supporting Figure 1. Effect of CCl4 and CCl4 /GW501516 treatment on Pparα and Pparγ expression in mouse liver.Supporting Figure 2.Quantification of Sirus Red staining in mouse liver sections. (PDF 143 kb) [file 2045-3701-2-34-S1.pdf]

SUPPLEMENTARY INFORMATION

Supporting Figure 1. *Effect of CCl4 and CCl4/GW501516 treatment on PPARα and PPARγ expression in mouse liver.*

qRT-PCR analysis of the expression of *Pparα* (top) and *Pparγ* (bottom) in liver of mice treated as indicated for 6 weeks. Control was the treatment with olive oil. WT, wild type mice; KO, PPARβ/δ-null mice. Means ± SEM (n=6). Note that the deletion of PPARβ/δ did not modify the expression of PPARα and PPARγ.

Supporting Figure 2. *Quantification of Sirus red staining in mouse liver sections.*

Sirus red staining of collagen fibers was quantified on sections (see Fig. 3A) from wild type (WT) and PPARβ/δ-null mice (KO). Quantification was performed with the NIS Elements software BR 3.0 (Nikon).

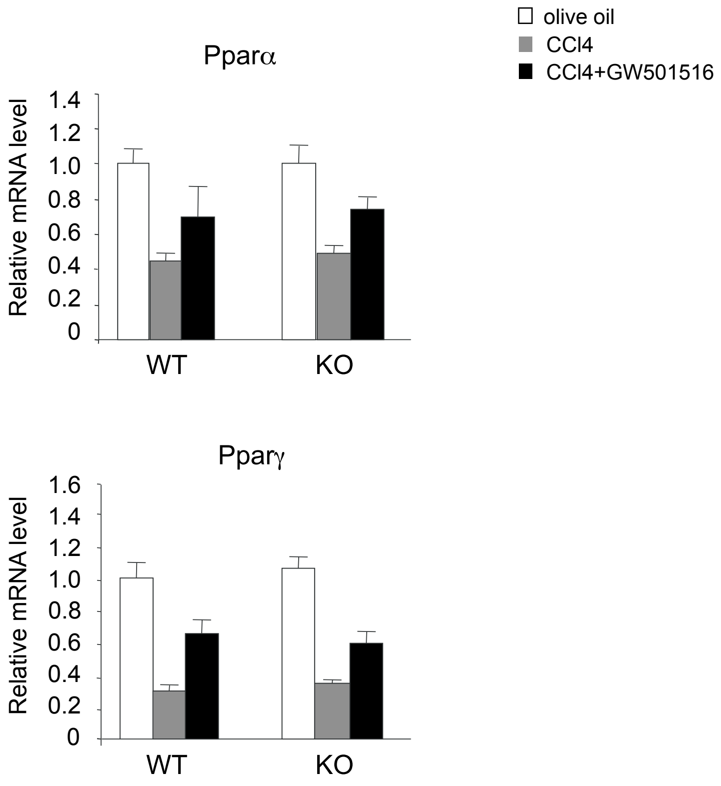

Supplementary Figure 1, Kostadinova R, et al

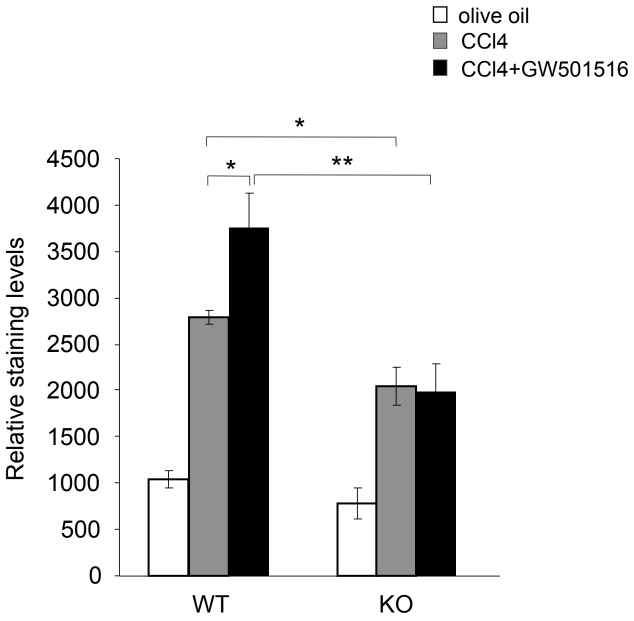

Supplementary Figure 2, Kostadinova R, et al
